# Insights into gemcitabine resistance in pancreatic cancer: association with metabolic reprogramming and *TP53* pathogenicity in patient derived xenografts

**DOI:** 10.1186/s12967-024-05528-6

**Published:** 2024-08-05

**Authors:** Mariam M. Konaté, Julia Krushkal, Ming-Chung Li, Li Chen, Yuri Kotliarov, Alida Palmisano, Rini Pauly, Qian Xie, P. Mickey Williams, Lisa M. McShane, Yingdong Zhao

**Affiliations:** 1 Biometric Research Program, Division of Cancer Treatment and Diagnosis, National Cancer Institute, National Institutes of Health, Rockville, MD 20850 USA; 2 Leidos Biomedical Research, Inc, Frederick National Laboratory for Cancer Research, Frederick, MD 21704 USA; 3 General Dynamics Information Technology (GDIT), Falls Church, VA 22042 USA

**Keywords:** Pancreatic adenocarcinoma, Gemcitabine, Intrinsic resistance, Acquired resistance, OXPHOS, Glycolysis, TP53

## Abstract

**Background:**

With poor prognosis and high mortality, pancreatic ductal adenocarcinoma (PDAC) is one of the most lethal malignancies. Standard of care therapies for PDAC have included gemcitabine for the past three decades, although resistance often develops within weeks of chemotherapy initiation through an array of possible mechanisms.

**Methods:**

We reanalyzed publicly available RNA-seq gene expression profiles of 28 PDAC patient-derived xenograft (PDX) models before and after a 21-day gemcitabine treatment using our validated analysis pipeline to identify molecular markers of intrinsic and acquired resistance.

**Results:**

Using normalized RNA-seq quantification measurements, we first identified oxidative phosphorylation and interferon alpha pathways as the two most enriched cancer hallmark gene sets in the baseline gene expression profile associated with intrinsic gemcitabine resistance and sensitivity, respectively. Furthermore, we discovered strong correlations between drug-induced expression changes in glycolysis and oxidative phosphorylation genes and response to gemcitabine, which suggests that these pathways may be associated with acquired gemcitabine resistance mechanisms. Thus, we developed prediction models using baseline gene expression profiles in those pathways and validated them in another dataset of 12 PDAC models from Novartis. We also developed prediction models based on drug-induced expression changes in genes from the Molecular Signatures Database (MSigDB)’s curated 50 cancer hallmark gene sets. Finally, pathogenic *TP53* mutations correlated with treatment resistance.

**Conclusion:**

Our results demonstrate that concurrent upregulation of both glycolysis and oxidative phosphorylation pathways occurs in vivo in PDAC PDXs following gemcitabine treatment and that pathogenic *TP53* status had association with gemcitabine resistance in these models. Our findings may elucidate the molecular basis for gemcitabine resistance and provide insights for effective drug combination in PDAC chemotherapy.

**Supplementary Information:**

The online version contains supplementary material available at 10.1186/s12967-024-05528-6.

## Introduction

Pancreatic cancer is a major public health concern as the third leading cause of cancer-related mortality in the United States and seventh worldwide [1]. There are two main subtypes of pancreatic cancer: pancreatic neuroendocrine tumor (PanNET) which accounts for < 5% of cases, and the more prevalent pancreatic ductal adenocarcinoma (PDAC) which accounts for ~ 90% of cases [2]. The disease is often diagnosed at an advanced stage so that prognosis for PDAC remains extremely poor with < 10% 5-year survival rate [3]. This is due, in part, to the fact that early symptoms are ambiguous and reliable early detection markers are lacking. Moreover, PDAC is believed to metastasize microscopically at an early stage [4]. Along with radiation and surgery, chemotherapy based on the pyrimidine antimetabolite gemcitabine (2’,2’-difluorodeoxycytidine) is the standard treatment for locally advanced and metastatic PDAC [5]. Gemcitabine causes apoptosis via multiple mechanisms, including the inhibition of DNA synthesis and deoxynucleotide metabolism, and the activation of pro-apoptotic caspase signaling [6]. However, in clinical practice, gemcitabine chemotherapy regimens confer modest survival benefits often accompanied by drug-associated adverse effects [7].

PDAC is notorious for chemoresistance to standard therapy, both intrinsic and acquired [4–6, 8]. Mechanisms related to the transport and metabolism of gemcitabine were first identified as causes of chemoresistance [6]. Moreover, PDAC tumors are most frequently driven by alterations in *KRAS*, *TP53*, *CDKN2A*, and *SMAD4* [9–12]. Furthermore, treatment promotes acquired resistance in as little as a few weeks [13]. The composition of the tumor microenvironment constitutes another barrier to drug efficacy due to stromal proliferation and reduced vascularization which prevent drug diffusion to the tumor, and immune suppression [4]. However, these processes cannot effectively be studied in vitro as cell line cancer models lack the immune components and stromal content needed to recapitulate tumor microenvironment. These shortcomings can be partly addressed by undertaking in vivo experiments in mice bearing xenografts from established cancer cell lines or patient-derived tumors. Multiple studies have established that patient-derived xenografts (PDXs) have greater translational potential than xenografted cancer cell lines which tend to be more divergent from the original tumor due to extended passaging in vitro [14–17]. Despite microenvironmental differences (i.e., human stroma and extracellular matrix in the original tumor compared to mouse-derived components in xenograft experiments), PDX models retain the three-dimensional architecture of the original tumor, as well as genetic characteristics and metastatic potential [17]. Therefore, the development of PDX models facilitates the implementation of drug response studies, and the potential identification of clinically relevant biomarkers of response and resistance [18–20].

In a recent study, Yang et al. constructed 66 pancreatic cancer PDX mouse models [21]. The PDXs were treated with gemcitabine for 21 days and the drug responses were evaluated by tumor growth inhibition (TGI%). The PDX models displayed a range of TGI% at 21 days of treatment with gemcitabine and were classified into four groups: sensitive, partial sensitive, partial resistant, and resistant by TGI%. To uncover molecular characteristics of gemcitabine-resistant and -sensitive models, multi-omics techniques were used to compare the molecular features of PDX tissues from the sensitive (*n* = 15) and resistant (*n* = 13) models [21]. The models classified as partial sensitive and partial resistant were not sequenced and were therefore not included in the multi-omics analysis by Yang et al. [21], nor in the present study.

The comparative transcriptional analysis described in the original study did not identify any significantly enriched pathways associated with gemcitabine sensitive and resistant phenotypes before treatment, and three pathways were found to be enriched after gemcitabine treatment with adjusted *p* < 0.125 [21]. This is likely due to the use of TPM (transcripts per million) data as input for the DESeq2 package differential expression analysis rather than raw read counts, which is improper as DESeq2 requires raw counts of sequencing reads or fragments as input [22, 23]. Therefore, in the present study, we set out to reprocess and reanalyze the PDAC PDX gene expression data produced by Yang et al. (referred to as the Yang dataset hereafter) using our validated pipeline to identify markers of intrinsic and acquired resistance to gemcitabine. The association between presence of pathogenic *TP53* mutations and gemcitabine response was also examined.

## Methods

Additional File 1: Figure S1 provides an overview of the steps of data collection and analyses included in this study.

### Data acquisition and preprocessing

For the Yang dataset, RNA-seq data were obtained from the Genome Sequence Archive (CRA002096). Fifty-six FASTQ files for 28 PDAC PDX models with available RNA-seq data were downloaded including both baseline and post gemcitabine treatment measurements. Then, FASTQ files were processed with the Frederick National Laboratory for Cancer Research’s Molecular Characterization Laboratory (i.e., MoCha) pipeline as described previously [24]. PDX mouse reads were removed from the raw FASTQ files using the bbsplit (bbtools v37.36) bioinformatic package (sourceforge.net/projects/bbmap/). The FASTQ files were mapped to the human transcriptome based on exon models from hg19 using Bowtie 2 (version 2.2.6) [25]. The resulting SAM files were converted to BAM format using samtools [26], and the transcriptomic coordinates from the BAM file were converted to the corresponding genomic (hg19) coordinates using RSEM (version 1.2.31) [27]. Gene level quantification was also performed with RSEM (version 1.2.31). The R package tximport was used to prepare gene level count data from RSEM output files, which provided a data matrix containing 28,109 genes.

To validate the initial findings based on baseline gene expression, an independent dataset of 12 PDAC PDX models generated by Novartis (Novartis Institutes for BioMedical Research PDX encyclopedia [NIBR PDXE]) [28] was extracted from the Bioconductor package Xenograft Visualization & Analysis (Xeva) [29]. This dataset is referred to as the Novartis dataset hereafter.

### Gemcitabine response

**Fig. 1:**
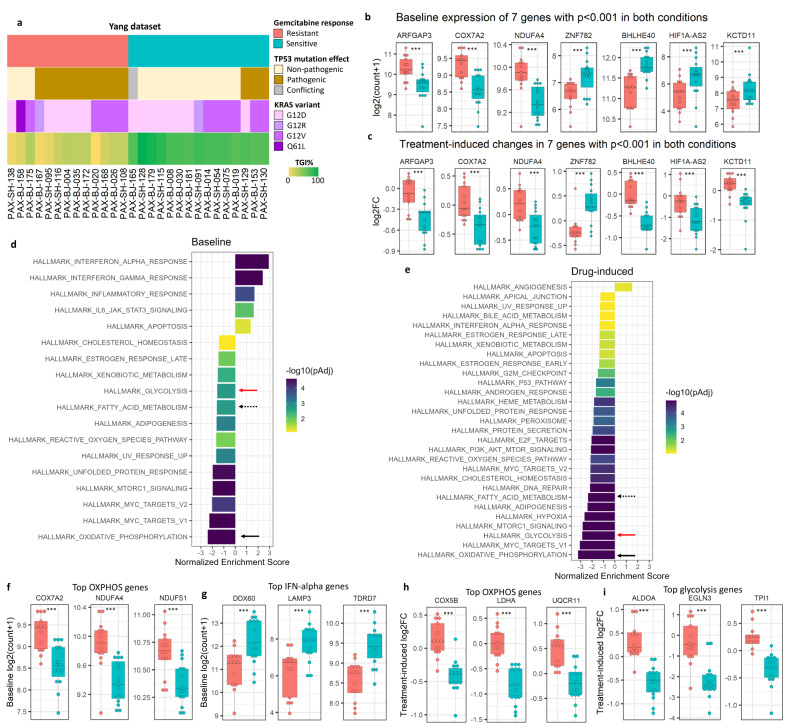
Phenotypic characteristics of the PDAC PDXs and gemcitabine response dependent molecular and gene set features. **(a)** Heatmap of phenotypic features for the 28 PDAC PDX models from the Yang dataset. **(b**,** c)** Baseline expression **b)** and drug-induced log2 fold-change (log2FC) **c)** of seven genes with *p* < 0.001 both at baseline and in drug-induced changes. **(d**,** e)** Significantly enriched pathways from GSEA in sensitive compared to resistant models at baseline **d)** and 21 days after start of treatment **e)** in sensitive compared to resistant models identified using multi-factor paired design in DESeq2. Benjamini-Hochberg adjusted *p* < 0.10. The x-axis represents the Normalized Enrichment Score (NES). Darker bars denote lower enrichment adjusted *p* values. Solid black arrows indicate oxidative phosphorylation (OXPHOS), solid red arrows indicate glycolysis, and dashed black arrows denote fatty acid metabolism. **(f**,** g)** Top three differentially expressed genes in the most enriched pathways at baseline: the OXPHOS pathway enriched in resistant models **f)** and the IFN-alpha pathway enriched in sensitive models **g)**. **(h**,** i)** Top three genes with greatest drug-induced change in expression from the OXPHOS pathway **h)** and the glycolysis pathway **i)**. ***p < 0.001

Of the 28 PDX models in the study by Yang et al. [21], 15 were deemed sensitive to gemcitabine and 13 were deemed resistant according to percent tumor growth inhibition (TGI%) at the conclusion of 21 days of treatment (Fig. 1a). Briefly, TGI% at 21 days was computed by Yang et al. using the following formula:

$\:TGI\%=\left[1-\:\frac{{T}_{21}\:-\:{T}_{0}}{{C}_{21}\:-\:{C}_{0}}\right]\:\times\:100$, where *T*_*21*_ and *C*_*21*_ are the average tumor volume on the 21st treatment day in the gemcitabine group and control group, respectively. *T*_*0*_ and *C*_*0*_ were the average tumor volume on day 0 in the gemcitabine group and control group, respectively. The models with TGI% > 100 at 21 days were labeled as sensitive and the models with TGI% < 50 at 21 days were labeled as resistant. These phenotypic labels for gemcitabine response were used for our differential expression analyses.

For the Novartis dataset, the standard treatment schedule was also 21 days and the response to gemcitabine was assessed by modified Response Evaluation Criteria in Solid Tumors (mRECIST) criteria as described by Gao et al. [28], that is by computing the change in tumor volume at 21 days compared to its baseline. Out of the 12 models, four were complete responders (CR: best response < − 95%) to gemcitabine and eight had progressive disease (PD: best response > 35%).

### Differential analysis, correlation analysis, and gene set enrichment analysis (GSEA)

Differential gene expression analysis was carried out using Bioconductor R package DESeq2 (version 1.36.0) [22] to identify molecular differences between gemcitabine sensitive and resistant models at baseline (i.e., prior to treatment). Furthermore, differences in drug-induced gene expression changes between sensitive and resistant models were also evaluated. For the latter analysis, we used multi-factor paired design in DESeq2 with PDX model (paired pre- and post-treatment) and gemcitabine response (sensitive and resistant) as the factors to identify genes for which gemcitabine treatment effects differed significantly between sensitive and resistant models while also controlling for model differences. Gene set enrichment analysis (GSEA) was performed with Bioconductor R package FGSEA (version 1.22.0) [30]. The DESeq2 differential expression results tables were sorted by test statistic and used as inputs for FGSEA along with the Molecular Signature Database (MSigDB)’s 50 cancer hallmark gene sets [31, 32]. Gene sets were considered significantly enriched if Normalized Enrichment Score |NES| > 1 and Benjamini-Hochberg false discovery rate (FDR) adjusted *p* < 0.10 [30]. To investigate the effects of gemcitabine treatment on gene expression, we also conducted a Spearman-rank correlation analysis between the drug-induced changes in gene expression and the continuous drug response, represented as TGI% at 21 days. We used the resulting test statistics as input for a pathway enrichment analysis using a software package developed in-house (available upon request). The analysis was performed on a curated set of 96 metabolic pathways that were previously defined in a study by Gaude and Frezza [33]. These pathways were used as the list of query gene sets. LS statistic was used to determine the significance of pathway enrichment [34, 35].

### Prediction model development and validation

Compound Covariate Predictor (CCP), Diagonal Linear Discriminant Analysis (DLDA), Near Centroid (NC), and Support Vector Machine (SVM) models were used to predict drug response from baseline gene expression data of 64 OXPHOS and glycolysis genes with differential drug-induced changes significantly correlated with TGI% at 21 days (reference [36] and references therein). The performance of the prediction models was evaluated using Leave-One-Out Cross Validation (LOOCV). In each round of LOOCV, features (genes) were re-selected according to criteria that they must be significantly differentially expressed between the sensitive and resistant classes at 0.05 significance level from t-test in order to be used for class prediction. The final prediction model using baseline data as input was validated on an independent dataset (Novartis dataset) that included 12 PDAC PDX models, their response to gemcitabine, and RNA-seq gene expression data [28, 29]. R package ComBat was applied to the validation data (Novartis dataset) using the Yang dataset as a reference to correct the batch effect [37]. We also developed least absolute shrinkage and selection operator (LASSO) and least angle regression (LARS) models to predict gemcitabine response, measured as binary response and as a continuous outcome (TGI%), respectively, using Yang’s dataset. The prediction utilized a treatment-induced expression matrix, with gene selection restricted to the 50 Cancer Hallmark pathways. By focusing on the perturbed gene expression within these pathways, our aim was to gain deeper insights into the mechanisms underlying resistance to gemcitabine. The use of continuous outcome data (TGI%) in the LARS model also provided an additional benefit of increased statistical power [38, 39]. The performance of these prediction models was also evaluated using Leave-One-Out Cross Validation (LOOCV). All prediction models were built using BRB-ArrayTools v4.6.2 [36].

### Annotation of TP53 and KRAS mutational status, and analysis of TP53 targets mediating its effects on glycolysis and OXPHOS

*TP53* and *KRAS* mutational information in the pancreatic tumor samples was obtained from the supplementary data of Yang et al. [21]. Here and below, we use *TP53* for the gene name, and p53 for its protein product; furthermore, we refer to a model as a combined representation of all tumor specimens (clinical and PDX-derived) which were derived from a single initial PDAC patient. The trend for observed associations between the *TP53* mutation status and response to gemcitabine was further interrogated using supplementary data of the Novartis dataset from Gao et al. [28]. In our analysis, we classified the *TP53* mutation status of each model into three categories (pathogenic, non-pathogenic, and conflicting) based on the category most indicative of pathogenicity of all *TP53* mutation variants reported for each model. Detailed information about *TP53* variant classification in the Yang and Novartis datasets is provided in **Additional File 2** and its accompanying legend. Briefly, information about functional effects of *TP53* mutation variants was obtained from the OncoKB and compared to the information from ClinVar, CiViC, and the Jackson Laboratory Clinical KnowledgeBase (JAX CKB) databases, and from biomedical publications. These additional data sources were in strong agreement with the OncoKB classification of the *TP53* variants analyzed in our study. Statistical association of the *TP53* mutation status with gemcitabine response in PDX models was tested using a two-sided Fisher’s exact test. All models in the Yang dataset carried *KRAS* variants that were classified as oncogenic by the OncoKB, and their pathogenicity was confirmed by biomedical literature sources.

To investigate molecular effects of *TP53* and its pathogenic variants on gemcitabine resistance, we analyzed the expression of 14 genes (*SLC2A1/GLUT1*,* SLC2A3/GLUT3*,* SLC2A4/GLUT4*,* C12orf5/TIGAR*,* HK2*,* SCO2*,* AIFM1/AIF*,* GLS2*,* HIF1A*,* PGM1*,* SLC1A3*,* SLC7A3*,* BBC3/PUMA*, and *RRAD*) whose products mediate *TP53* effects on glycolysis and OXPHOS [40–44]. We used univariate Spearman-rank correlation analysis of DESeq2-normalized expression count each of genes with TGI%, as well as linear regression modeling (lm) of TGI%, using *TP53* mutation status and expression of each target gene as predictor variables.

## Results

### Transcriptional differences associated with gemcitabine resistance in PDAC xenografts tend to be acquired rather than intrinsic

The analysis steps of our study are schematized in Additional File 1: Figure S1. First, we sought to identify molecular markers of intrinsic resistance to gemcitabine in PDAC. To this end, we carried out differential expression analysis comparing baseline expression of 28,109 genes in 13 gemcitabine-resistant xenograft models to 15 gemcitabine-sensitive models using DESeq2. One hundred and nine genes were differentially expressed between gemcitabine sensitive and resistant models at baseline with unadjusted *p* < 0.001 (Additional File 3: Table S1). Out of these, 22 genes were significantly differentially expressed between the two gemcitabine response groups after FDR adjustment at 5%. Ten genes fulfilled both |log2FC| ≥ 1 and FDR adjusted *p* < 0.05 requirements; *GCAT*, *HHIPL2*, *IL33*, and *KLF15* had higher baseline expression in resistant models, while *DDX60*, *ESAM*, *IL12RB1*, *LOC100132077*, *MYBPC1*, and *STARD9* had higher baseline expression in sensitive models (Additional File 1: Figs. S2a and S2b; Additional File 3: Table S1).

Next, we examined differences in gene expression trajectories associated with treatment in gemcitabine-sensitive and in gemcitabine-resistant models. This was achieved by using multi-factor paired design in DESeq2 (Additional File 1: Fig. S2c). This approach specifically identified genes with different drug-induced effects between gemcitabine sensitive and resistant models. Out of 28,109 genes, we found 1,206 genes with significant differences between drug-induced changes in expression in sensitive and resistant models and unadjusted *p* < 0.001. We found 442 genes to have significantly higher drug-induced expression changes in gemcitabine-sensitive models and 143 genes to have significantly lower drug-induced expression changes in gemcitabine-sensitive models with |log2FC| ≥ 1 and adjusted *p* < 0.05 (Additional File 3: Table S2). There was no overlap between significant hits identified at baseline and those differentially induced by drug treatment in sensitive and resistant models at 5% FDR, but seven genes were differentially expressed both at baseline and in drug-induced data with unadjusted *p* < 0.001 (Fig. 1b and c). *ARFGAP3*, *COX7A2*, and *NDUFA4* were more highly expressed in resistant models at baseline and drug-induced changes increased this effect; *ZNF782* was more highly expressed in sensitive models at baseline and had drug-induced upregulation in sensitive models. In contrast, *BHLHE40*, *HIF1A-AS2*, and *KCTD11* were more highly expressed in sensitive models at baseline and were significantly downregulated only in sensitive models with treatment (Fig. 1b and c).

Taken together, these findings suggest that at the transcriptional level, baseline gemcitabine-sensitive and resistant xenograft models were more variable in their gene expression so that fewer genes reached significance when comparing these two groups at baseline. We observed a large number of differences in drug-induced transcriptional changes between the two response groups, suggesting distinct and consistent patterns of differential expression in gemcitabine-resistant and sensitive models with a 21-day treatment. Expression of these differentially modulated genes may be associated with acquired gemcitabine resistance (Additional File 1: Fig. S2).

### Baseline and treatment-induced gemcitabine resistance is associated with enrichment of metabolic pathway genes

In the Yang dataset (Fig. 1a), gene set enrichment analysis (GSEA) revealed that genes differentially expressed at baseline and differentially modulated with treatment in gemcitabine-resistant models compared to sensitive models were significantly enriched in multiple metabolism-related pathways such as OXPHOS, fatty acid metabolism, and glycolysis (Fig. 1d and e; Additional File 3: Tables S3 and S4). More fatty acid metabolism and glycolysis genes were associated with gemcitabine resistance in drug-induced differential expression data than at baseline, as evidenced by the higher magnitude NES and lower *p* values (Fig. 1d and e). Furthermore, genes with drug-induced upregulation in resistant models were significantly enriched in various additional pathways, including DNA repair, MYC targets, mTORC1 signaling, and p53 pathway. In contrast, gemcitabine-sensitive models were found to be strongly associated with expression of interferon alpha and gamma response genes at baseline (Fig. 1d and Additional File 3: Table S3), and drug-induced upregulation of angiogenesis genes (Fig. 1e and Additional File 3: Table S4). These results indicate that gemcitabine treatment modulates many pathways in resistant models compared to sensitive models. The top three OXPHOS genes most highly expressed at baseline in resistant models compared to sensitive models were *COX7A2*, *NDUFA4*, and *NDUFS1* (Fig. 1f). Interestingly, *COX7A2* and *NDUFA4* were further upregulated in resistant models with treatment as stated above and illustrated in Fig. 1c. The top three genes from the IFN-alpha response pathway most highly expressed in sensitive models compared to resistant models were *DDX60*, *LAMP3*, and *TDRD7* (Fig. 1g). Moreover, *COX5B*, *LDHA* (also in the glycolysis pathway), and *UQCR11* in the OXPHOS pathway; and *ALDOA*, *EGLN3*, and *TPI1* in the glycolysis pathway were the genes with greatest drug-induced expression changes in their respective pathways (Fig. 1h and i). Comprehensive lists of genes driving gene set enrichments can be found in the *leadingEdge* column of Additional File 3: Tables S3 and S4.

### Drug-induced changes in glycolysis and OXPHOS genes correlate with tumor growth inhibition (TGI%) at 21 days of treatment

**Fig. 2:**
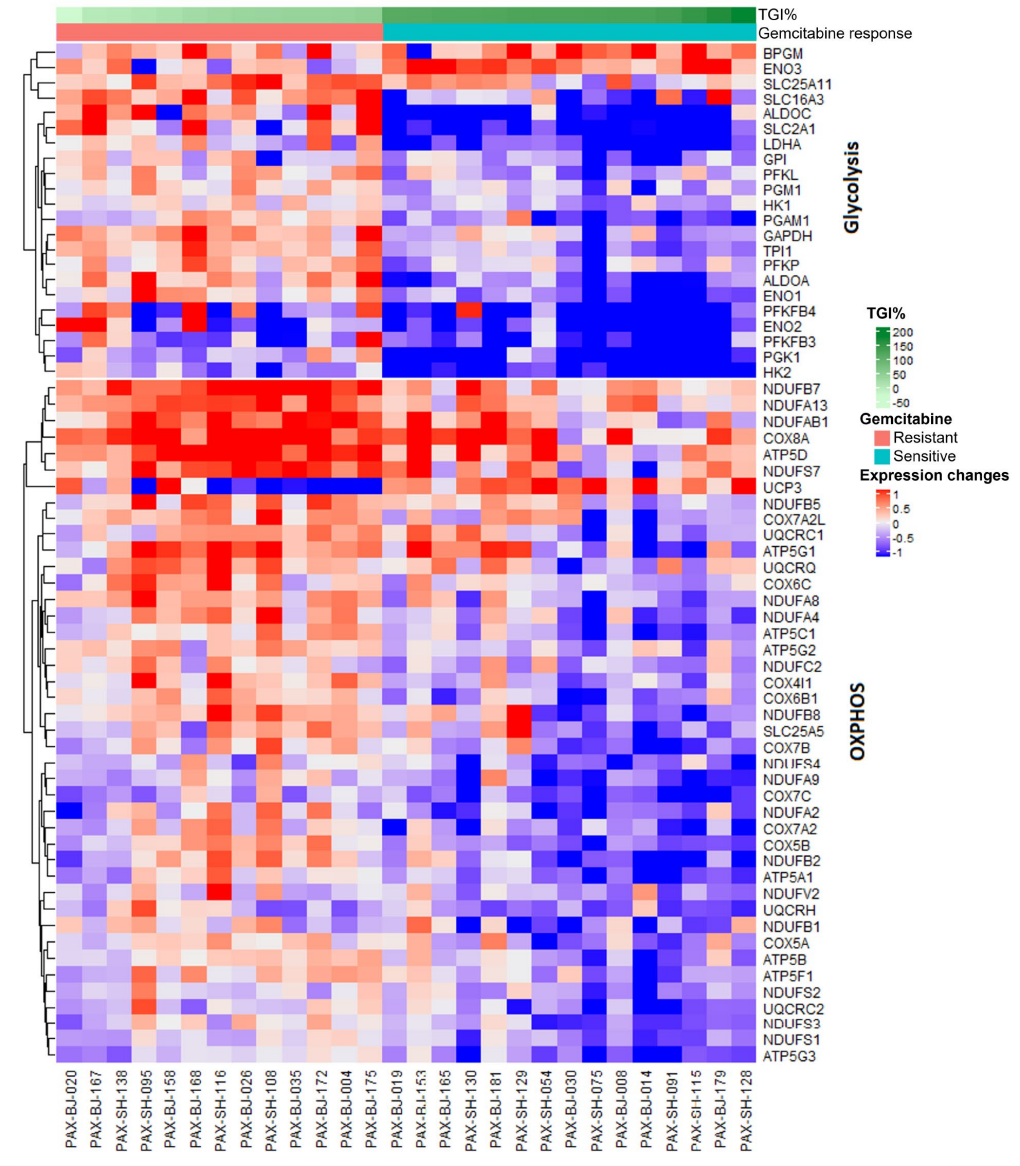
Heatmap of genes whose drug-induced expression changes are significantly correlated with TGI% of gemcitabine (*p* < 0.05) in the glycolysis and OXPHOS pathways. The heatmap displays expression changes using the Z-score of the log2FC between post-treatment expression and baseline expression

In addition to differential expression analysis with DESeq2 using categorical drug response outcome in the PDX models, Spearman-rank correlation analyses were performed to examine associations between drug-induced gene expression changes and continuous outcome tumor growth inhibition (TGI% at 21 days) as defined in the paper by Yang et al. [21]. Our goal was to identify genes for which drug-induced expression changes in the sensitive models differed from those in the resistant models. Among 4253 genes in 50 MSigDB cancer hallmark pathways, 127 genes were significantly correlated with TGI% at 21 days (adjusted *p* value < 0.05, Additional File 3: Table S5). GSEA was also performed on a manually-curated set of 96 human metabolic pathways containing 1453 unique genes from a study by Gaude and Frezza [33]. The set of genes correlated with TGI% was mainly enriched in glycolysis and gluconeogenesis, as well as OXPHOS (Additional File 3: Table S6). In the glycolysis and gluconeogenesis pathway, *TPI1*, *ENO1*, *LDHA*, *HK2*, *GAPDH*, *ALDOA*, *SLC2A1*, *ENO2*, *PGK1*, *PGAM1* were the genes significantly correlated with TGI% (adjusted *p* values < 0.05). In the OXPHOS pathway, *COX5B*, *ATP5C1*, *ATP5A1*, and *NDUFA8* were the genes significantly correlated with TGI% (adjusted *p* values < 0.05). These results are summarized as a heatmap in Fig. 2 and in Additional File 3: Table S7.

### Baseline expression of OXPHOS and glycolysis genes differentially modulated with treatment can predict gemcitabine response

**Fig. 3:**
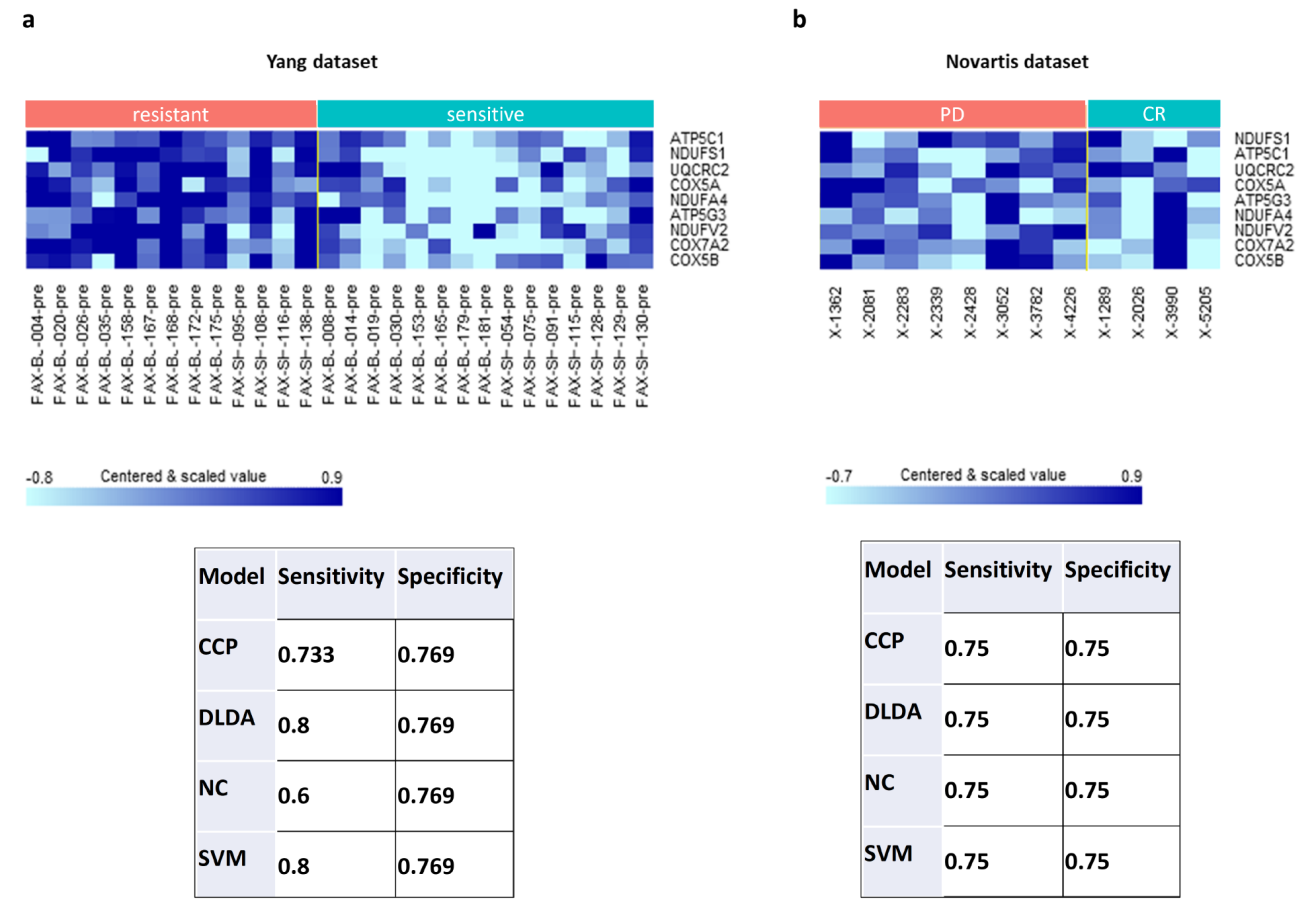
Validation of predictive 9-gene baseline expression signature for binary response to gemcitabine. **(a**,** b)** Heatmaps of expression of 9-gene signature in the prediction models of binary response using baseline expression in the Yang dataset **a)** and validated in the Novartis dataset **b)**, and performance in both datasets. CR: complete response to gemcitabine (i.e., sensitive); PD: progressive disease (i.e., resistant to gemcitabine)

Through our analysis of drug-induced changes and tumor growth inhibition (TGI%) at 21 days, we noted the significant enrichment of glycolysis and OXPHOS metabolic pathways among genes with drug-induced upregulation in resistant models. We proposed to build prediction models of drug response using baseline expression levels and restricting the pool of predictive features (i.e., genes) to genes from these two pathways with drug-induced changes in expression significantly correlated with TGI% at 21 days. Our hypothesis was that genes whose expression levels change in response to a drug may have predictive value even at baseline as they may be involved in the drug’s mechanism of action or may be part of a pathway or network that is affected by the drug. We started the modeling process by selecting the 22 genes from the glycolysis pathway and 42 genes from the OXPHOS pathway whose drug-induced change in expression showed significant association with TGI% at 21 days at *p* < 0.05. We then developed prediction models including CCP, DLDA, NC, and SVM, based on the baseline expression profile of these 64 genes. The final models included nine genes, all of which belong to the OXPHOS pathway, and their expression patterns in the Yang and Novartis datasets are detailed in Additional File 4 and illustrated in Fig. 3. We evaluated the models’ performance in the Yang dataset using LOOCV and presented the sensitivity and specificity, for all four models in Fig. 3a. Among them, DLDA and SVM exhibited the best performance, with a sensitivity of 0.80 and specificity of 0.77 in the training set (i.e., Yang dataset). To further validate the model, we used the 12 PDX PDAC models from Novartis that either had a complete response (CR) to gemcitabine (i.e., sensitive) or progressive disease (PD) (i.e., resistant) [28]. The validation set produced comparable results, with a sensitivity of 0.75 and specificity of 0.75 (Fig. 3b).

### Prediction modeling of gemcitabine response using drug-induced expression profile of cancer hallmark pathway genes

**Fig. 4:**
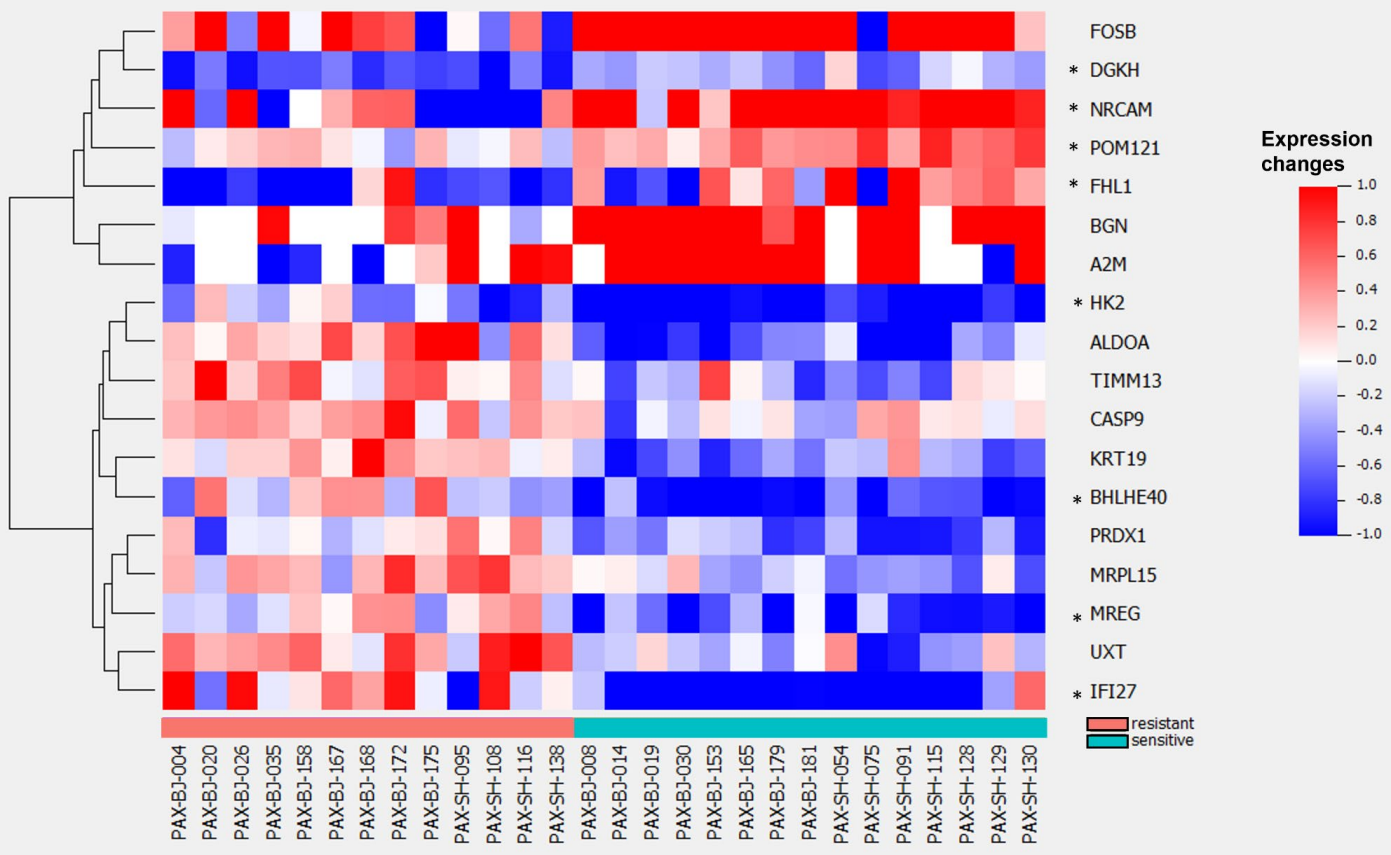
LASSO model of binary response to gemcitabine using drug-induced transcriptional differences between sensitive and resistant models in cancer hallmark genes. Heatmap of 18 genes from MSigDB’s cancer hallmark gene sets in a LASSO model predicting binary outcome based on drug-induced changes. Asterisks (*) denote genes also appearing in the LARS model predicting continuous outcome TGI% for gemcitabine

To further investigate drug resistant mechanisms, we developed a model to predict the binary outcome of drug response—i.e., sensitive or resistant to gemcitabine—using LASSO regression on drug-induced changes of 4,253 genes from the 50 MSigDB cancer hallmark gene sets [32]. The LOOCV demonstrated appreciable model performance with sensitivity at 0.93 and specificity at 1.0. Eighteen genes were selected by the LASSO in the full data set (Fig. 4): *A2M*, *ALDOA*, *BGN*, *BHLHE40*, *CASP9*, *DGKH*, *FHL1*, *FOSB*, *HK2*, *IFI27*, *KRT19*, *MREG*, *MRPL15*, *NRCAM*, *POM121*, *PRDX1*, *TIMM13*, *UXT* (Additional File 5).

We also developed a LARS model to predict continuous TGI% at 21 days using the drug-induced change in expression of the same 4,253 MSigDB cancer hallmark genes (Additional File 1: Fig. S3 and Additional File 6). Eight genes appeared in both models based on either continuous outcome or binary outcome: *BHLHE40*, *DGKH*, *FHL1*, *HK2*, *IFI27*, *MREG*, *NRCAM*, *POM121*.

### Presence of pathogenic TP53 variants is associated with gemcitabine resistance

**Fig. 5:**
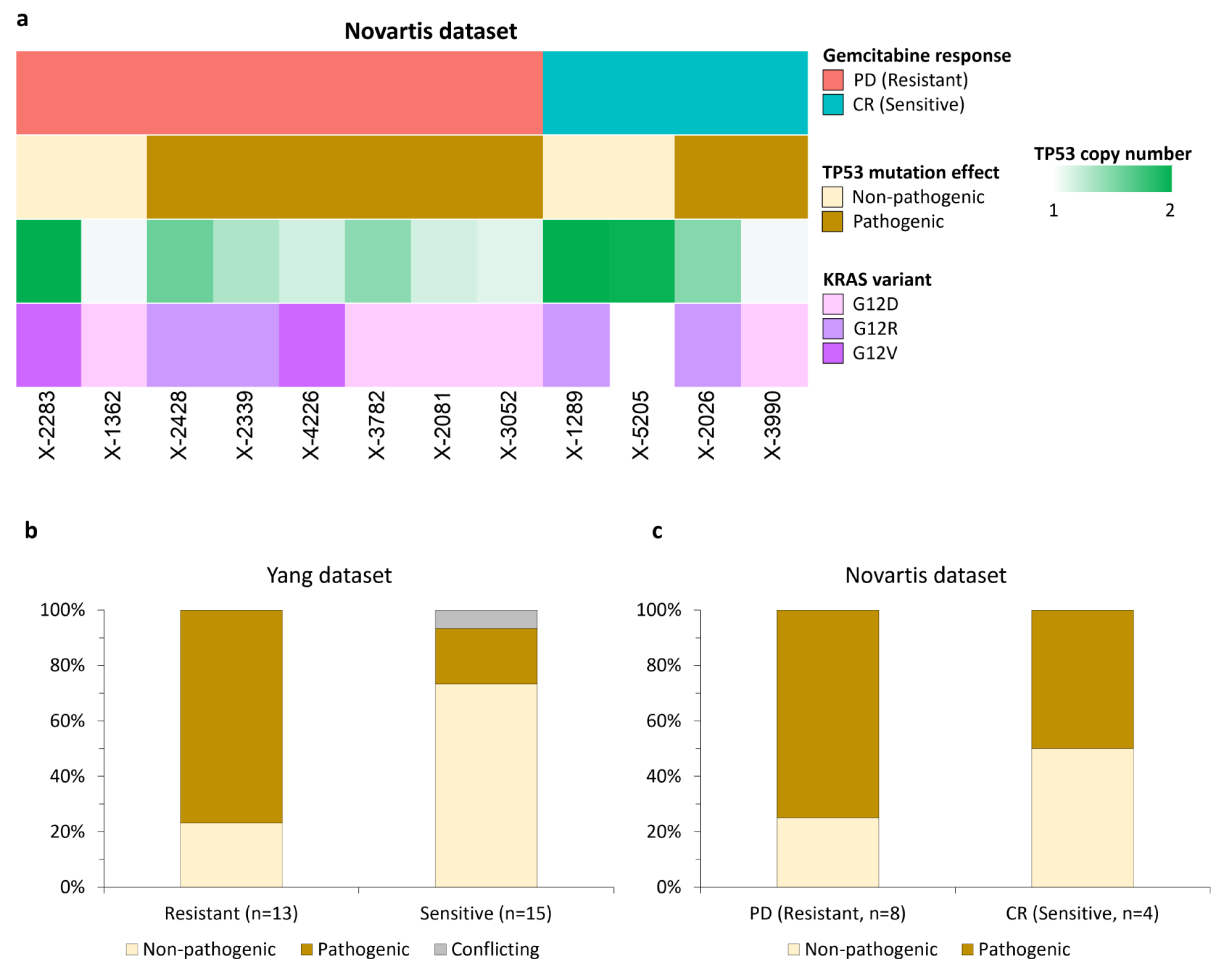
Significance of *TP53* variants in two pancreatic cancer patient-derived xenograft datasets. **(a)** Heatmap of phenotypic features for the 12 PDAC PDX models from the Novartis dataset. *TP53* mutational information is from the supplementary data of Yang et al.; *TP53* status of each model was classified as non-pathogenic, pathogenic, or having conflicting evidence based on annotations from OncoKB, ClinVar, CIViC, and/or the Jackson Laboratory Clinical KnowledgeBase (JAX CKB). **(b)** Yang dataset (graphical view of TP53 mutation data is shown in Fig. 1a); 13 models were resistant to gemcitabine and 15 models were sensitive as determined by the evaluation of percentage tumor growth inhibition (TGI%). Fisher’s exact test for proportion of non-pathogenic and pathogenic *TP53* categories between gemcitabine resistant and sensitive models, **p = 0.0067. **(c)** Novartis dataset; eight models were resistant to gemcitabine and four models were sensitive as determined by the modified RECIST criteria defined in the paper by Gao et al. Fisher’s exact test for proportion of non-pathogenic and pathogenic *TP53* categories between gemcitabine resistant and sensitive models, *p* = 0.55

Since *TP53*, the second most frequently mutated gene in PDAC, controls metabolic networks, cell death, and transcriptional regulation in normal pancreatic cells and the rewiring of these pathways in PDAC tumors [40, 45, 46], we investigated whether pathogenic mutations in *TP53* were associated with response to gemcitabine. Additional File 1 lists TP53 mutation categories and gemcitabine response status of the PDX models in the Yang and Novartis datasets; the frequency of different categories of *TP53* variants among PDX gemcitabine responder and non-responder samples in both datasets are presented in Additional File 3: Table S8. In the Yang dataset, those samples that did not carry pathogenic *TP53* variants were significantly more likely to be sensitive to gemcitabine (*p* = 0.0067). In the Novartis dataset (Fig. 5a), there was a lower prevalence of pathogenic *TP53* variants in complete responders (2 out of 4, or 50%) compared to patients with progressive disease (6 out of 8, or 75%), but the association did not reach statistical significance (*p* = 0.55). These data are presented in Fig. 5b and c.

p53 effects on glycolysis and mitochondrial respiration are mediated by several molecular targets [47, 48]. We observed a strong negative association of TGI% at 21 days with drug-induced expression changes of two such target genes, glucose transporter *SCL2A1*/*GLUT1* and hexokinase 2 *HK2* (Spearman ρ = -0.69 and − 0.73, respectively, with FDR adjusted *p* < 0.05; Additional file 3: Table S7) [42, 43]. We further employed linear regression analysis of TGI% at 21 days using both *TP53* mutation status and expression (or drug-induced change in expression) of each of the 14 p53 target genes mediating its effects on glycolysis and OXPHOS as predictor variables (Additional file 3: Table S9). This analysis revealed the significant association between *TP53* mutation status and TGI% at 21 days, even after adjusting for baseline expression or drug-induced expression changes of the 14 target genes (FDR adjusted *p* < 0.05); and that gemcitabine-induced changes in expression of both *SLC2A1* and *HK2* were significantly associated with TGI% after adjusting for *TP53* mutation status (FDR adjusted *p* = 0.004 for both genes). Drug-induced *SCL2A1* and *HK2* expression changes were strikingly different between models with pathogenic vs. non-pathogenic *TP53*, with significant downregulation (*p* < 0.01) in non-pathogenic models (Additional File 1: Figs. S4a and S4b).

## Discussion

PDAC is a highly aggressive malignancy with poor patient prognosis. PDAC tumors undergo extensive molecular reprogramming, resulting in increased glycolysis and elevated mitochondrial function [49–52], among multiple other metabolic changes. In the present work, we have demonstrated not only a wide-ranging transcriptional reprogramming with gemcitabine treatment (Additional File 1: Fig. S2 and Additional File 3: Table S2) that differed between sensitive and resistant models, but also differences in baseline expression of OXPHOS and inflammatory pathways associated with resistant and sensitive models, respectively (Fig. 1; Additional File 3: Tables S3 and S4). *NDUFA4* and *COX7A2* are both components of the cytochrome c oxidase and were shown in multiple studies to control metabolic regulation between OXPHOS and glycolysis depending on oxygen availability [53–55]. In line with these findings, we observed the baseline overexpression and drug-induced upregulation of *NDUFA4* and *COX7A2* in resistant PDXs (Fig. 1b and c).

Glycolysis and OXPHOS occur on a tight balance to provide cells with energy in the form of adenosine triphosphate (ATP) [44]. In normal cells, the availability of oxygen makes OXPHOS the more efficient option [44, 56]. The transcriptional analysis in the original study by Yang et al. had not identified any significantly enriched pathways associated with intrinsic resistance to gemcitabine [21]. In our pathway analysis of the PDAC PDXs, resistant models were mainly enriched in OXPHOS genes at baseline and with treatment (as indicated by the negative NES; Fig. 1d and e). These results suggest that OXPHOS may play a role in intrinsic resistance to gemcitabine. While few existing studies link OXPHOS to gemcitabine resistance, dependency on mitochondrial OXPHOS has been linked to the presence of pancreatic cancer stem cells (CSCs) and dormant, therapy-evading tumor cells [57, 58]. CSCs represent a small but tumorigenic subset of cells in some tumors [59, 60]. A recent study revealed that pancreatic tumors could be stratified according to their OXPHOS activity and expression of mitochondrial respiratory complex I [49]. Furthermore, targeting OXPHOS with phenformin, an inhibitor of mitochondrial respiratory complex I, potentiated the activity of gemcitabine in pancreatic cancer in vitro and in vivo [49], providing further support for potential importance of OXPHOS in gemcitabine resistance.

Consistent with our findings implicating glycolytic metabolism in acquired gemcitabine resistance, glycolysis has been widely implicated with chemoresistance in the literature [61]. Unrestricted PDAC tumor growth promotes hypoxia, which in turn, activates glycolysis via the induction of the transcription factor hypoxia-inducible factor 1 (HIF1) [62–64]. Xu et al. demonstrated glycolysis-associated gemcitabine resistance in BxPC-3 pancreatic cancer cell lines and derived xenografts via the upregulation of *HIF1A* by *HIF1A-AS1* [65]. *HIF1-AS2*, another antisense RNA of *HIF1A*, is a hypoxia-induced oncogene [66, 67], which under hypoxic conditions upregulates *HIF1A*, promotes cisplatin and gemcitabine resistance, and suppresses p53 activity [66, 68, 69]. In our analysis, *HIF1A-AS2* was strongly and significantly downregulated in sensitive models after treatment (Fig. 1c).

Our observation that both glycolysis and OXPHOS were strongly enriched in gemcitabine resistant PDAC PDX models after treatment (Fig. 1) is in line with accounts of hybrid OXPHOS/glycolysis phenotype associated with metabolic plasticity, chemoresistance, and metastasis [49, 70]. This could also be explained by the presence of heterogeneous cell subpopulations with distinct metabolic priorities [57]. Fujiwara-Tani et al. established a gemcitabine-resistant pancreatic cancer cell line derived from MIA-PaCa-2 and demonstrated that in the absence of gemcitabine the resistant cell line relied on OXPHOS for ATP generation [71]. However, upon exposure to gemcitabine, OXPHOS was suppressed in favor of glycolysis, and mitochondrial-associated reactive oxygen species (ROS) were decreased [71]. In the present study, the enrichment of OXPHOS and glycolysis at baseline and in drug-induced changes in resistant PDAC models was highly significant (*p* < 0.001), however these pathways were not identified in the original transcriptional analysis of the Yang dataset [21]. This can likely be explained by the fact that the authors used TPM normalized data as input for DESeq2 analysis rather than the required raw counts, which has been shown by our group and others to be improper [22–24]. DESeq2 has been designed for the use of raw RNA-seq read counts [22, 23]. DESeq2 assumes a negative binomial distribution for RNA-seq count data. TPM normalized data does not follow a negative binomial distribution and therefore is not appropriate to be used as input for DESeq2 normalization and downstream analyses such as differential expression analysis. Our group established recently in the first comparative study of RNA-seq data quantification measures conducted on PDX models that normalized count data are the preferred quantification measure for between-sample analysis of RNA-seq data generated from tumors grown in PDX models. We also demonstrated that further data transformations or normalizations on TPM-level data are not able to resolve potential issues inherent in TPM quantification [24].

For translational purposes, that is to be able to predict patient response prior to treatment administration, accurate prediction models on baseline expression data would be useful. We used knowledge of drug-induced pathway enrichment results to build well-performing CCP, DLDA, NC, and SVM prediction models on baseline expression data by restricting the pool of selectable features to 64 glycolysis and OXPHOS genes (Additional File 4**).** We recognize that using genes identified from differential drug-induced expression changes to build baseline prediction models could introduce information leakage due to potential correlations between drug-induced changes and baseline data. To further validate the model in an unbiased approach, we applied the established model to an independent dataset from Novartis and demonstrated appreciable performance in this validation set (Fig. 3).

*KRAS* and *TP53* are the most frequently mutated PDAC genes [45, 72]. All models in the Yang dataset had oncogenic *KRAS* mutations (Fig. 1a). Pathogenic *TP53* status had significant association with gemcitabine resistance in Yang’s data and a similar trend in the Novartis dataset (Fig. 5b and c; Additional File 3: Table S8), albeit not significant, possibly due to the small size of the Novartis dataset (*n* = 12). We also observed a trend for *TP53* deletion in resistant Novartis models (Fig. 5a), consistent with *TP53* loss through both pathogenic mutation and deletion. Yang’s data did not contain copy number information. Our findings confirm associations of *TP53* loss with worse human patient PDAC outcomes and with increased tumor growth and gemcitabine resistance in mouse models [73–76], although one clinical trial of PDAC patients reported an opposite effect on adjuvant gemcitabine efficacy [76].

Wild type p53 regulates metabolism by inhibiting glycolysis, promoting the TCA cycle and OXPHOS, limiting flux through the pentose phosphate pathway (PPP), inhibiting *de novo* serine biosynthesis, promoting fatty acid oxidation and transport, regulating amino acid metabolism, and helping cells survive under nutrient starvation [40–42]. The majority of its pathogenic variants result in the loss of p53 function, leading to activation of glycolysis, impaired OXPHOS, increase in ROS, inhibition of autophagy, and increased hypoxia [40–42, 72, 75]. *SLC2A1* and *HK2*, promote glycolysis, are inhibited by the wild type p53, and are upregulated in cancer cells with pathogenic *TP53* [42–44]. Association of their upregulation with TGI% is consistent with the upregulation of glycolysis in gemcitabine resistant models (Additional File 1: Fig. S4; Additional File 3: Table S7), as well as with higher prevalence of pathogenic *TP53* variants and upregulation of several other metabolic pathways in gemcitabine resistant PDX PDAC models observed in our study, although metabolic changes in PDAC could be influenced by multiple factors and may not be driven solely by the loss of *TP53*.

## Conclusion

In conclusion, we provide important cross-validated findings that may inform the development of gemcitabine-based combination therapies. Further studies are needed to ascertain whether our results obtained from PDX RNA-seq data would be confirmed in patient data.

### Electronic supplementary material

Below is the link to the electronic supplementary material.

Supplementary Material 1: **Additional File 1: Figure S1**. Schematic illustration of data analyses. **Figure S2**. Baseline and drug-induced differential gene expression analysis between gemcitabine-sensitive and resistant models. **Figure S3**. Least Angle Regression (LARS) model of continuous response to gemcitabine (TGI%) using drug-induced transcriptional differences between sensitive and resistant models in cancer hallmark genes. **Figure S4**. Expression of p53 target genes *SLC2A1/GLUT1* and *HK2* at baseline and post-treatment stratified by gemcitabine response and *TP53* variant type.

Supplementary Material 2: **Additional File 2**: *TP53* mutation categories and gemcitabine response status of the PDX models in the Yang and Novartis datasets.

Supplementary Material 3: **Additional File 3: Table S1**. Differentially expressed genes at baseline in gemcitabine-sensitive vs. gemcitabine-resistant models with unadjusted p < 0.001. **Table S2**. Significant differences in drug-associated changes in gene expression (i.e., deltas post-treatment vs. baseline) between sensitive and resistant models. **Table S3**. Significantly enriched gene sets between gemcitabine sensitive and resistant PDAC models at baseline. **Table S4**. Significantly enriched gene sets for genes with different drug-induced expression profiles between gemcitabine sensitive and resistant PDAC models. **Table S5**. Genes from MSigDB’s cancer hallmark gene sets with drug-induced expression changes significantly correlated with TGI%. **Table S6**. Significantly enriched pathways in 96 metabolic pathway gene sets. **Table S7**. Genes in glycolysis and OXPHOS pathways for which gemcitabine-induced expression changes were significantly correlated with TGI%. **Table S8**. Prevalence of *TP53* mutational categories stratified by gemcitabine response status in the Yang and Novartis datasets. Fisher’s exact text p values are indicated for each dataset. **Table S9**. Significance of the effect of *TP53* pathogenic status effect and differences in expression or expression changes on TGI% at 21 days for p53 target genes adjusted for *TP53* effect or gene effect.

Supplementary Material 4: **Additional File 4**: Details of prediction models using baseline gene expression profile.

Supplementary Material 5: **Additional File 5**: LASSO model for prediction of binary response based on drug-induced changes.

Supplementary Material 6: **Additional File 6**: Least Angle Regression model for prediction of continuous response based on drug-induced gene expression changes.

## Data Availability

The datasets analyzed in this manuscript were acquired from the Genome Sequence Archive (CRA002096) for the Yang dataset and the Bioconductor package Xeva (https://www.bioconductor.org/packages/release/bioc/html/Xeva.html) for the Novartis dataset.

## Abbreviations

ATP: Adenosine triphosphate
CCP: Compound covariate predictor
CR: Complete response
CSC: Cancer stem cell
dCTP: Deoxycytidine triphosphate
DLDA: Diagonal linear discriminant analysis
ES: Enrichment score
FC: Fold-change
FPKM: Fragments per kilobase of transcript per million
GSEA: Gene set enrichment analysis
JAX CKB: Jackson Laboratory Clinical Knowledgebase
LARS: Least angle regression
LASSO: Least absolute shrinkage and selection operator
LOOCV: Leave-one-out cross validation
mRECIST: Modified response evaluation criteria in solid tumors
MSigDB: Molecular signature database
NC: Near Centroid
NES: Normalized enrichment score
OXPHOS: Oxidative phosphorylation
PanNET: Pancreatic neuroendocrine tumor
PD: Progressive disease
PDAC: Pancreatic ductal adenocarcinoma
PDX: Patient-derived xenograft
PPP: Pentose phosphate pathway
ROS: Reactive oxygen species
SVM: Support vector machine
TGI: Tumor growth inhibition
TPM: Transcripts per million
