# Supplementary material for: Insights into gemcitabine resistance in pancreatic cancer: association with metabolic reprogramming and TP53 pathogenicity in patient derived xenografts
Source: J Transl Med. 2024 Aug 5;22:733. doi: 10.1186/s12967-024-05528-6 (PMC11301937; doi:10.1186/s12967-024-05528-6)
Supplement: Supplementary file 1 — Supplementary Material 1: Additional File 1: Figure S1. Schematic illustration of data analyses. Figure S2. Baseline and drug-induced differential gene expression analysis between gemcitabine-sensitive and resistant models. Figure S3. Least Angle Regression (LARS) model of continuous response to gemcitabine (TGI%) using drug-induced transcriptional differences between sensitive and resistant models in cancer hallmark genes. Figure S4. Expression of p53 target genes SLC2A1/GLUT1 and HK2 at baseline and post-treatment stratified by gemcitabine response and TP53 variant type. [file 12967_2024_5528_MOESM1_ESM.docx]

**Additional File 1: Figure S1.** Schematic illustration of data analyses. Graphical summary of PDAC PDX data created with BioRender.

PDAC PDX data (Yang *et al.*, 2021)


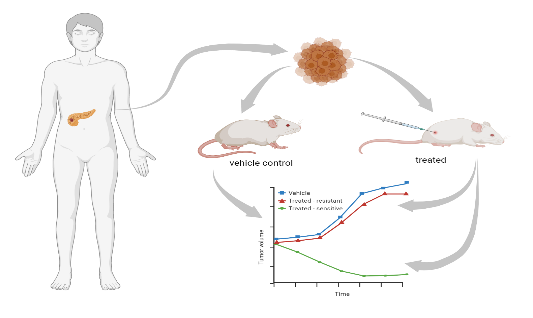


Associate *TP53* mutation
with binary drug response in Yang and Novartis datasets

Prediction models CCP/DLDA/NC/SVM for
binary drug response outcome

LOOCV to evaluate performance

pre-treatment data

DE analysis (DESeq2):
15 sensitive vs. 13 resistant models

Use these 64 gene symbols to build prediction models on pre-treatment data

GSEA (FGSEA) on 50 cancer hallmark gene sets (MSigDB)

64 genes significantly correlated with TGI% in the top two most significantly enriched gene sets

Glycolysis and OXPHOS

GSEA on 96 metabolic gene sets

(Gaude and Frezza, 2016)

Prediction models (LASSO/LARS) for

binary and continuous drug response using 4253 genes in the 50 cancer hallmark gene sets

LOOCV to evaluate performance

Validate the established 9-gene full prediction model from the Yang dataset on the Novartis dataset

GSEA (FGSEA) on 50 cancer hallmark gene sets (MSigDB)

DE analysis (DESeq2):
15 sensitive vs. 13 resistant models

Spearman-rank correlative analysis with continuous drug response TGI%

drug-induced change data

Re-process FASTQ files using FNLCR MoCha RNA-seq pipeline

Count matrix of 28109 genes

FASTQ

RSEM

tximport

raw count

**Additional File 1: Figure S2. Baseline and drug-induced differential gene expression analysis between gemcitabine-sensitive and resistant models using DESeq2. (a)** Volcano plots for differential gene expression between sensitive and resistant models at baseline. **(b)** Differentially expressed genes at baseline between sensitive and resistant models with |log2FC| ≥ 1 and FDR adjusted *p* < 0.05. ****p* < 0.001. **(c)** Volcano plot for drug-induced differential gene expression between sensitive and resistant models. Criteria for significant differential expression are |log2 FC| ≥ 1 (i.e., |FC| ≥ 2) and FDR adjusted *p* < 0.05. A total of 28109 genes were analyzed.

**
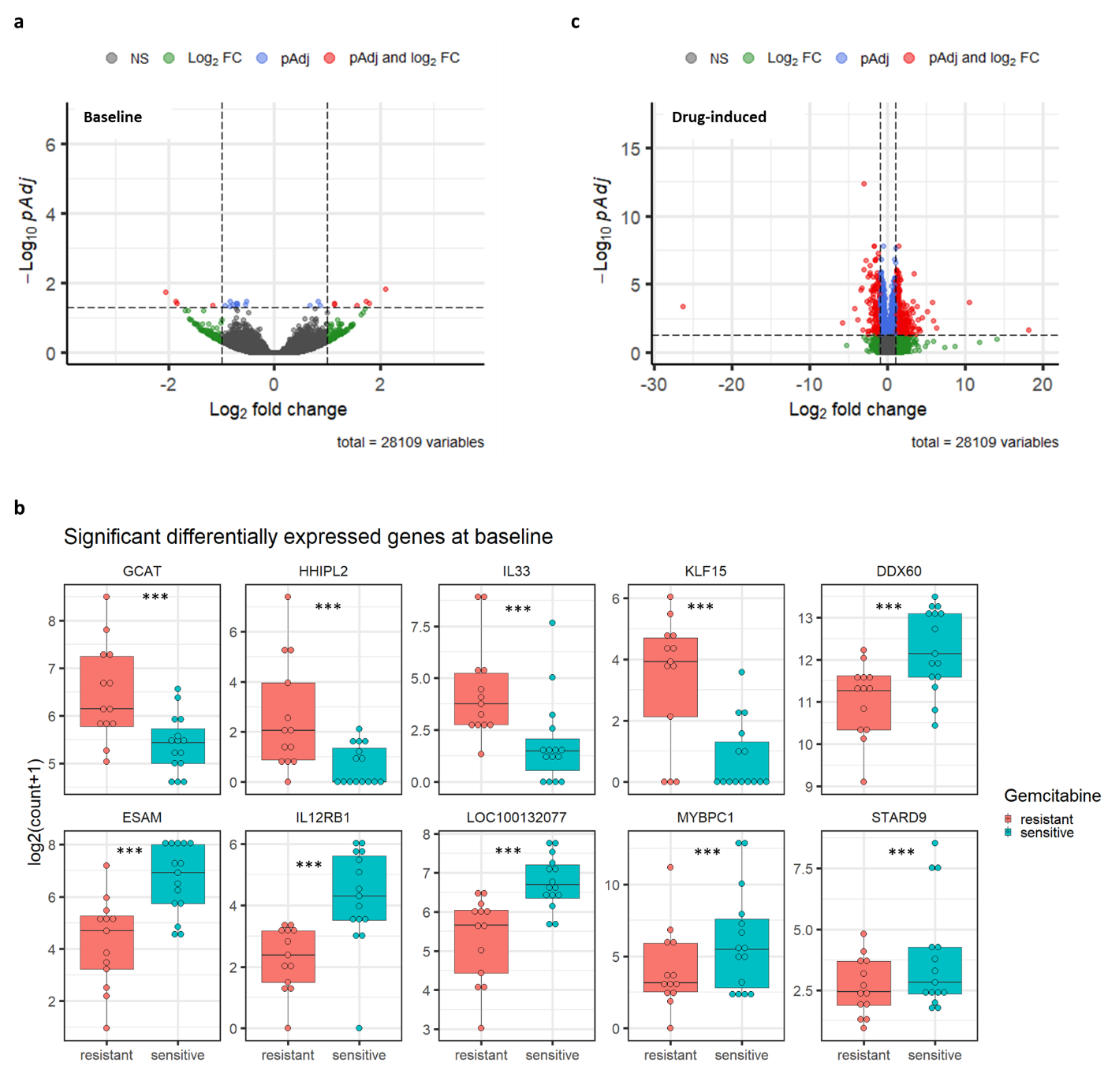
**

**Additional File 1: Figure S3**. **Least Angle Regression (LARS) model of continuous response to gemcitabine (TGI%) using drug-induced transcriptional differences between sensitive and resistant models in cancer hallmark genes. (a)** Scatter plot of predicted versus actual TGI% of gemcitabine from leave-one-out cross validation (LOOCV) of a least angle regression (LARS) model. **(b)** Heatmap of 25 genes from MSigDB’s 50 cancer hallmark gene sets in a LARS model predicting continuous outcome TGI% based on drug-induced changes.


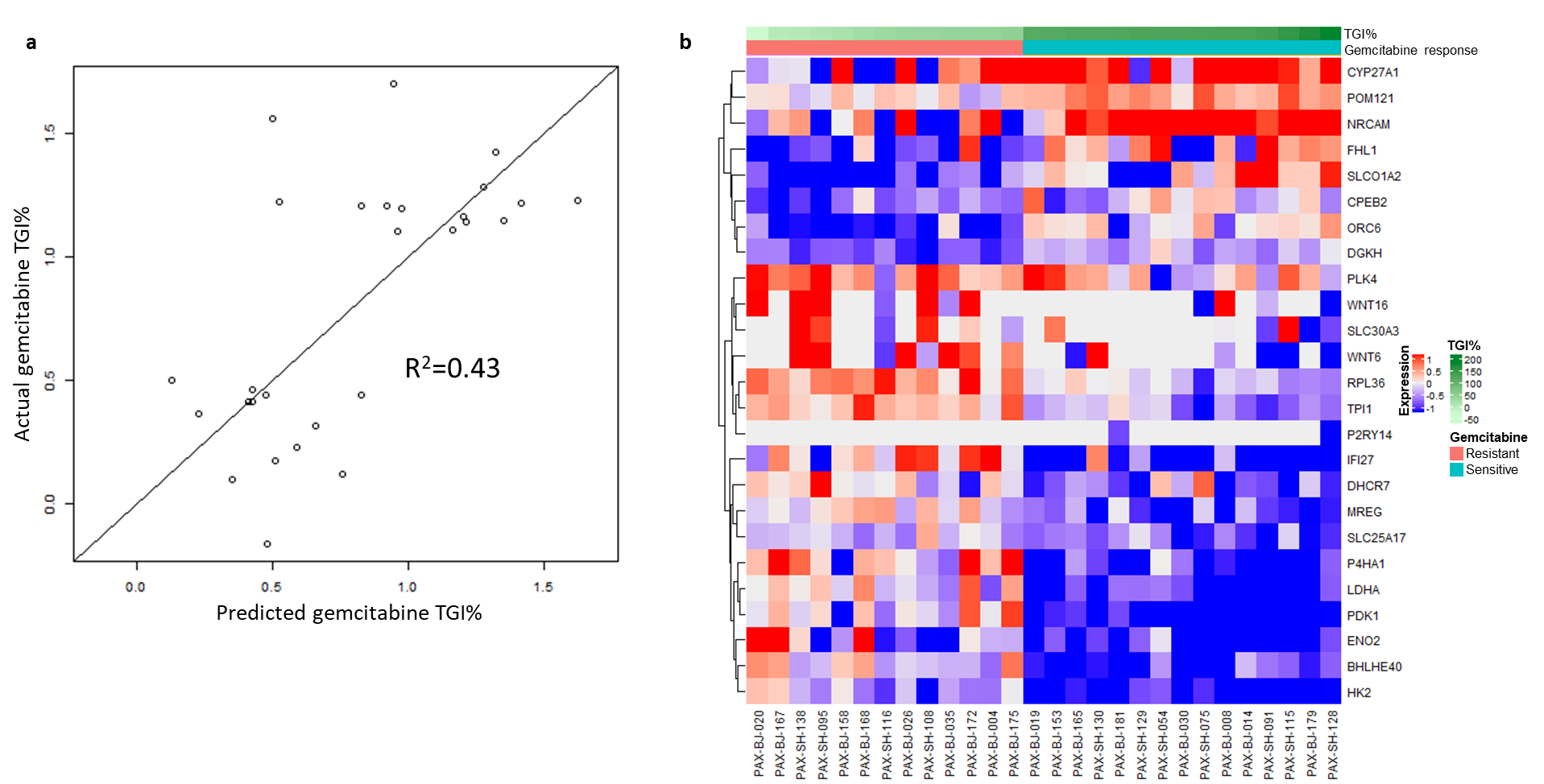


**Additional File 1: Figure S4.** Expression of p53 target genes *SLC2A1/GLUT1* and *HK2* at baseline (i.e., ‘pre’) and post-treatment stratified by gemcitabine response and *TP53* variant type. ***p* < 0.01, **p* < 0.05; n.s., not significant.

**
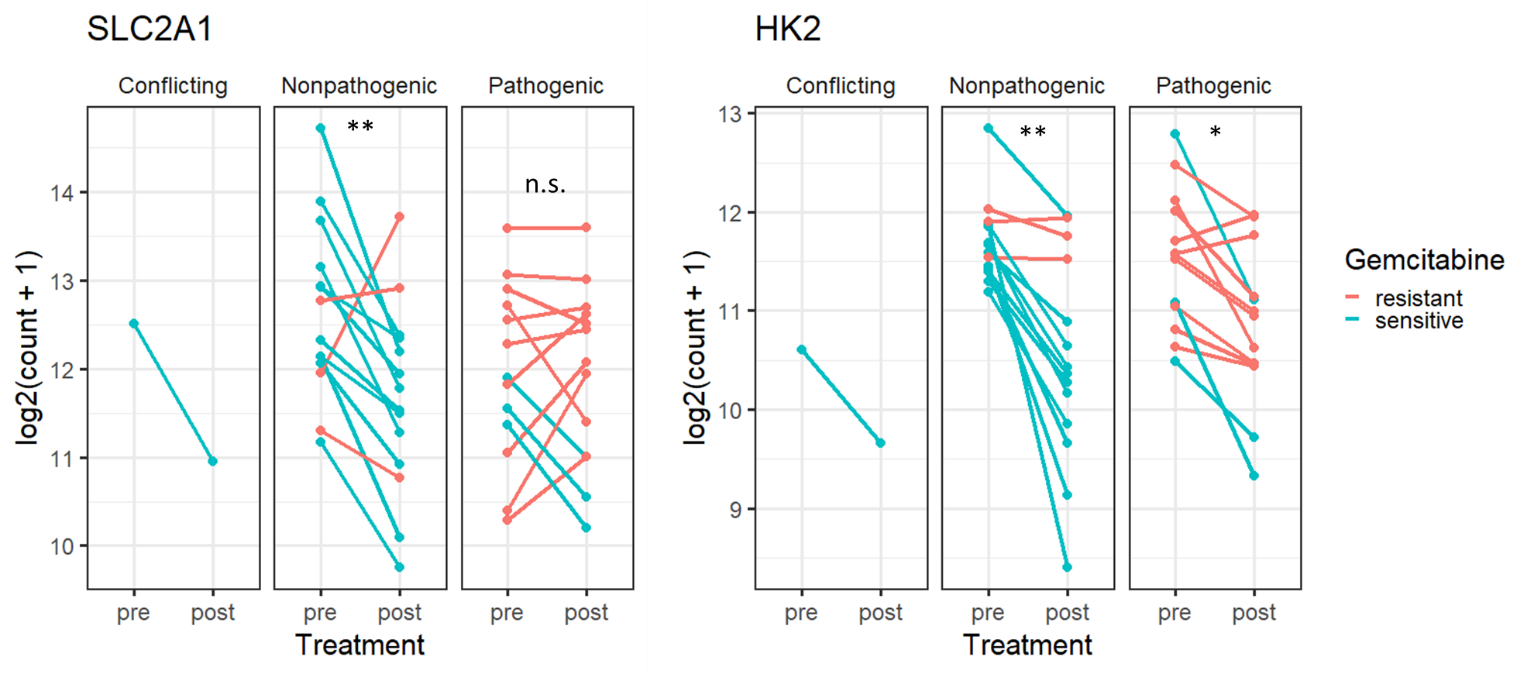
**
