# Supplementary material for: Insights into gemcitabine resistance in pancreatic cancer: association with metabolic reprogramming and TP53 pathogenicity in patient derived xenografts
Source: J Transl Med. 2024 Aug 5;22:733. doi: 10.1186/s12967-024-05528-6 (PMC11301937; doi:10.1186/s12967-024-05528-6)
Supplement: Supplementary file 2 — Supplementary Material 2: Additional File 2: TP53 mutation categories and gemcitabine response status of the PDX models in the Yang and Novartis datasets. [file 12967_2024_5528_MOESM2_ESM.docx]

**Additional File 2.** *TP53* mutation categories and gemcitabine response status of the PDX models in the Yang and Novartis datasets.

Information about functional effects of *TP53* mutation variants was obtained from the OncoKB and compared to the information from ClinVar, CiViC, and the Jackson Laboratory Clinical KnowledgeBase (JAX CKB) databases, and from biomedical publications. These additional data sources were in strong agreement with the OncoKB classification of the *TP53* variants analyzed in our study. In both Yang and Novartis datasets, all splice variants, premature gain of a stop codon (*), or frameshift changes (fs) were assigned to the pathogenic category, regardless of the availability of their annotation in external databases. All *TP53* variants which had Oncogenic or Likely Oncogenic Alteration, and/or Loss-of-function, Likely Loss-of-function, or Likely Gain-of-function Mutation Effect reported in OncoKB were assigned to the pathogenic category. The non-pathogenic *TP53* status was assigned to those samples that did not have any *TP53* variants or carried only the common germline polymorphism P72R. The conflicting category was assigned to the samples which carried only the nonsynonymous variant S215N that had conflicting evidence according to ClinVar and other resources. WT: wild type.

**A. *TP53* mutational category and gemcitabine response status of each tumor model in the Yang dataset (Yang, et al., 2021)**

Each tumor model was assigned a combined *TP53* mutational category status based on combined mutation data from all individual PDX samples and primary tumor samples for that model. If any tumor model had any PDX or primary tumor sample with at least one pathogenic *TP53* variant, that model was assigned to the pathogenic category, and information about its pathogenic variants is provided. For all other models which did not have any pathogenic *TP53* variants in any of their samples, information is provided about their *TP53* variants that were assigned non-pathogenic or conflicting status.

| **Tumor model** | **Gemcitabine response** | ***TP53* variant** | **Category** | **Variant class from *Yang et al.*** | **OncoKB Alteration** | **OncoKB Mutation Effect** | **Comment** | **References for pathogenicity determination** |
| --- | --- | --- | --- | --- | --- | --- | --- | --- |
| PAX-BJ-008 | Sensitive | **P72R** | Non-pathogenic | nonsynonymous SNV |  |  | Common germline polymorphism | (Barnoud, Parris and Murphy, 2019; Doffe, et al., 2021; Labuschagne, Zani and Vousden, 2018) |
| PAX-BJ-014 | Sensitive | **WT** | Non-pathogenic |  |  |  |  |  |
| PAX-BJ-019 | Sensitive | **WT** | Non-pathogenic |  |  |  |  |  |
| PAX-BJ-030 | Sensitive | **P72R** | Non-pathogenic | nonsynonymous SNV |  |  | Common polymorphism | (Barnoud, Parris and Murphy, 2019; Doffe, et al., 2021; Labuschagne, Zani and Vousden, 2018) |
| PAX-BJ-153 | Sensitive | **E258K** | Pathogenic | nonsynonymous SNV | Likely Oncogenic | Likely Loss-of-function | Model also has the common polymorphism P72R |  |
| PAX-BJ-165 | Sensitive | **S215N** | Conflicting | nonsynonymous SNV |  |  | Model also has the common polymorphism P72R | (Landrum, et al., 2018) |
| PAX-BJ-179 | Sensitive | **P72R** | Non-pathogenic | nonsynonymous SNV |  |  | Common germline polymorphism | (Barnoud, Parris and Murphy, 2019; Doffe, et al., 2021; Labuschagne, Zani and Vousden, 2018) |
| PAX-BJ-181 | Sensitive | **P72R** | Non-pathogenic | nonsynonymous SNV |  |  | Common germline polymorphism | (Barnoud, Parris and Murphy, 2019; Doffe, et al., 2021; Labuschagne, Zani and Vousden, 2018) |
| PAX-SH-054 | Sensitive | **WT** | Non-pathogenic |  |  |  |  |  |
| PAX-SH-075 | Sensitive | **P72R** | Non-pathogenic | nonsynonymous SNV |  |  | Common germline polymorphism | (Barnoud, Parris and Murphy, 2019; Doffe, et al., 2021; Labuschagne, Zani and Vousden, 2018) |
| PAX-SH-091 | Sensitive | **P72R** | Non-pathogenic | nonsynonymous SNV |  |  | Common germline polymorphism | (Barnoud, Parris and Murphy, 2019; Doffe, et al., 2021; Labuschagne, Zani and Vousden, 2018) |
| PAX-SH-115 | Sensitive | **WT** | Non-pathogenic |  |  |  |  |  |
| PAX-SH-128 | Sensitive | **WT** | Non-pathogenic |  |  |  |  |  |
| PAX-SH-129 | Sensitive | **R273H** | Pathogenic | nonsynonymous SNV | Oncogenic | Loss-of-function | Model also has the common polymorphism P72R | (Chakravarty, et al., 2017; Klemke, et al., 2021; Patterson, et al., 2016; Thijssen, et al., 2021) |
| PAX-SH-130 | Sensitive | **V173M** | Pathogenic | nonsynonymous SNV |  |  | Model also has the common polymorphism P72R | (Baroni, et al., 2004) |
| PAX-BJ-004 | Resistant | **R175H** | Pathogenic | nonsynonymous SNV | Oncogenic | Loss-of-function |  | (Chakravarty, et al., 2017; Kim, et al., 2021; Klemke, et al., 2021; Patterson, et al., 2016) |
| PAX-BJ-020 | Resistant | **R273C** | Pathogenic | nonsynonymous SNV | Likely Oncogenic | Likely Loss-of-function | Model also has the common polymorphism P72R | (Chakravarty, et al., 2017; Patterson, et al., 2016) |
| PAX-BJ-026 | Resistant | **E294X** | Pathogenic | stopgain |  |  | Model also has the common polymorphism P72R |  |
| PAX-BJ-035 | Resistant | **7578555 C->T** | Pathogenic | splice variant |  |  |  |  |
| PAX-BJ-158 | Resistant | **P72R** | Non-pathogenic | nonsynonymous SNV |  |  | Common germline polymorphism | (Barnoud, Parris and Murphy, 2019; Doffe, et al., 2021; Labuschagne, Zani and Vousden, 2018) |
| PAX-BJ-167 | Resistant | **S202fs** | Pathogenic | frameshift deletion |  |  | Model also has the common polymorphism P72R |  |
| PAX-BJ-168 | Resistant | **R282W** | Pathogenic | nonsynonymous SNV |  |  | Model also has the common polymorphism P72R | (Klemke, et al., 2021; Zhang, et al., 2016) |
| PAX-BJ-172 | Resistant | **G245S** | Pathogenic | nonsynonymous SNV | Oncogenic | Loss-of-function | Model also has the common polymorphism P72R | (Chakravarty, et al., 2017; Llovet, et al., 2017; Patterson, et al., 2016) |
| PAX-BJ-175 | Resistant | **P72R** | Non-pathogenic | nonsynonymous SNV |  |  | Common germline polymorphism | (Barnoud, Parris and Murphy, 2019; Doffe, et al., 2021; Labuschagne, Zani and Vousden, 2018) |
| PAX-SH-095 | Resistant | **R213*** | Pathogenic | stopgain |  |  | Model also has the common polymorphism P72R |  |
| PAX-SH-108 | Resistant | **V272L** | Pathogenic | nonsynonymous SNV | Likely Oncogenic | Likely Loss-of-function |  | (Chakravarty, et al., 2017; Landrum, et al., 2018) |
| PAX-SH-116 | Resistant | **R273H, V272L** | Pathogenic | nonsynonymous SNV | Oncogenic (R273H); Likely Oncogenic (V272L) | Loss-of-function (R273H); Likely Loss-of-function (V272L) | Model also has the common polymorphism P72R | (Chakravarty, et al., 2017; Klemke, et al., 2021; Landrum, et al., 2018; Patterson, et al., 2016) |
| PAX-SH-138 | Resistant | **WT** | Non-pathogenic |  |  |  |  |  |

**B. *TP53* mutational category and gemcitabine response status of the PDX samples in the Novartis dataset (Gao, et al., 2015)**

CR, complete response; PD, progressive disease, corresponding to sensitive and resistant categories, respectively.

| **Sample** | **Gemcitabine response** | ***TP53* variant** | **Category** | **Annotation by**  **Gao *et al.*** | **OncoKB Alteration** | **OncoKB Mutation Effect** | **Details from Gao *et al.*** | **Reference for pathogenicity determination** |
| --- | --- | --- | --- | --- | --- | --- | --- | --- |
| X-1289 | CR | **WT** | Non-pathogenic |  |  |  |  |  |
| X-2026 | CR | **E349*** | Pathogenic | MutKnownFunctional |  |  | 1.000 |  |
| X-3990 | CR | **P151H** | Pathogenic | MutNovel | Likely Oncogenic | Likely Loss-of-function | 0.987 | (Chakravarty, et al., 2017; Patterson, et al., 2016) |
| X-5205 | CR | **WT** | Non-pathogenic |  |  |  |  |  |
| X-1362 | PD | **WT** | Non-pathogenic |  |  |  |  |  |
| X-2081 | PD | **H214R** | Pathogenic | MutLikelyFunctional | Likely Oncogenic | Likely Loss-of-function | 0.989 | (Chakravarty, et al., 2017; Patterson, et al., 2016) |
| X-2283 | PD | **WT** | Non-pathogenic |  |  |  |  |  |
| X-2339 | PD | **R175H** | Pathogenic | MutKnownFunctional | Oncogenic | Loss-of-function | 0.995 | (Chakravarty, et al., 2017; Kim, et al., 2021; Klemke, et al., 2021; Patterson, et al., 2016) |
| X-2428 | PD | **E204*** | Pathogenic | MutKnownFunctional |  |  | 1.000 |  |
| X-3052 | PD | **R273H** | Pathogenic | MutKnownFunctional | Oncogenic | Loss-of-function | 1.000 | (Chakravarty, et al., 2017; Klemke, et al., 2021; Patterson, et al., 2016) |
| X-3782 | PD | **Y220C** | Pathogenic | MutKnownFunctional | Oncogenic | Loss-of-function | 1.000 | (Chakravarty, et al., 2017; Patterson, et al., 2016) |
| X-4226 | PD | **Y220C** | Pathogenic | MutKnownFunctional | Oncogenic | Loss-of-function | 0.992 | (Chakravarty, et al., 2017; Patterson, et al., 2016) |

**References**

Barnoud, T., Parris, J.L.D. and Murphy, M.E. Common genetic variants in the TP53 pathway and their impact on cancer. *J Mol Cell Biol* 2019;11:578-585.

Baroni, T.E.*, et al.* A global suppressor motif for p53 cancer mutants. *Proc Natl Acad Sci U S A* 2004;101:4930-4935.

Chakravarty, D.*, et al.* OncoKB: A Precision Oncology Knowledge Base. *JCO Precis Oncol* 2017;2017.

Doffe, F.*, et al.* Identification and functional characterization of new missense SNPs in the coding region of the TP53 gene. *Cell Death Differ* 2021;28:1477-1492.

Gao, H.*, et al.* High-throughput screening using patient-derived tumor xenografts to predict clinical trial drug response. *Nat Med* 2015;21:1318-1325.

Kim, M.P.*, et al.* Oncogenic KRAS Recruits an Expansive Transcriptional Network through Mutant p53 to Drive Pancreatic Cancer Metastasis. *Cancer Discov* 2021;11:2094-2111.

Klemke, L.*, et al.* The Gain-of-Function p53 R248W Mutant Promotes Migration by STAT3 Deregulation in Human Pancreatic Cancer Cells. *Front Oncol* 2021;11:642603.

Labuschagne, C.F., Zani, F. and Vousden, K.H. Control of metabolism by p53 - Cancer and beyond. *Biochim Biophys Acta Rev Cancer* 2018;1870:32-42.

Landrum, M.J.*, et al.* ClinVar: improving access to variant interpretations and supporting evidence. *Nucleic Acids Res* 2018;46:D1062-D1067.

Llovet, P.*, et al.* A novel TP53 germline inframe deletion identified in a Spanish series of Li-fraumeni syndrome suspected families. *Fam Cancer* 2017;16:567-575.

Patterson, S.E.*, et al.* The clinical trial landscape in oncology and connectivity of somatic mutational profiles to targeted therapies. *Hum Genomics* 2016;10.

Thijssen, R.*, et al.* Intact TP-53 function is essential for sustaining durable responses to BH3-mimetic drugs in leukemias. *Blood* 2021;137:2721-2735.

Yang, G.*, et al.* Integrative Genomic Analysis of Gemcitabine Resistance in Pancreatic Cancer by Patient-derived Xenograft Models. *Clin Cancer Res* 2021;27:3383-3396.

Zhang, Y., Coillie, S.V., Fang, J.Y. and Xu, J. Gain of function of mutant p53: R282W on the peak? *Oncogenesis* 2016;5:e196.
