# Supplementary material for: Insights into gemcitabine resistance in pancreatic cancer: association with metabolic reprogramming and TP53 pathogenicity in patient derived xenografts
Source: J Transl Med. 2024 Aug 5;22:733. doi: 10.1186/s12967-024-05528-6 (PMC11301937; doi:10.1186/s12967-024-05528-6)
Supplement: Supplementary file 4 — Supplementary Material 4: Additional File 4: Details of prediction models using baseline gene expression profile. [file 12967_2024_5528_MOESM4_ESM.docx]

**Additional File 4.** Details of prediction models using baseline gene expression profile.

**A.** Composition of classifier. Class 1: resistant; Class 2: sensitive.

|  | **Genes** | **Parametric p-value** | **t-value** | **% CV support** | **Geometric mean of intensities in class 1** | **Geometric mean of intensities in class 2** | **Fold-change** |
| --- | --- | --- | --- | --- | --- | --- | --- |
| 1 | *COX5B* | 0.0452155 | 2.104 | 43 | 626.88 | 514.26 | 1.22 |
| 2 | *NDUFV2* | 0.0286014 | 2.318 | 89 | 796.14 | 539.41 | 1.48 |
| 3 | *COX5A* | 0.0269533 | 2.345 | 96 | 396.45 | 275.79 | 1.44 |
| 4 | *ATP5G3* | 0.0190571 | 2.5 | 100 | 1198.46 | 952.47 | 1.26 |
| 5 | *ATP5C1* | 0.0065092 | 2.958 | 100 | 2247.63 | 1438.65 | 1.56 |
| 6 | *UQCRC2* | 0.0037489 | 3.184 | 100 | 2600.35 | 1866.01 | 1.39 |
| 7 | *NDUFS1* | 0.0008835 | 3.755 | 100 | 1832.4 | 1498.53 | 1.22 |
| 8 | *NDUFA4* | 0.0002628 | 4.22 | 100 | 1074.45 | 750.41 | 1.43 |
| 9 | *COX7A2* | 0.0001823 | 4.359 | 100 | 712.95 | 421.59 | 1.69 |

Prediction rule from the linear predictors: The prediction rule is defined by the inner sum of the weights (*w_i_*) and expression (*x_i_*) of significant genes. The expression is the log ratios for dual-channel data and log intensities for single-channel data. A sample is classified to the class resistant if the sum is greater than the threshold; that is, $\sum_{i} w_{i}x_{i}>threshold$

The thresholds are 274.273 for the Compound Covariate predictor (CCP), 283.737 for the Diagonal Linear Discriminant predictor (DLDA), and 37.175 for the Support Vector Machine predictor.

**B.** Gene weights.

|  | **Genes** | **Compound Covariate Predictor** | **Diagonal Linear Discriminant Analysis** | **Support Vector Machines** |
| --- | --- | --- | --- | --- |
| 1 | *ATP5C1* | 2.9584 | 1.9524 | 0.5583 |
| 2 | *ATP5G3* | 2.5 | 2.7077 | 0.2235 |
| 3 | *COX5A* | 2.3447 | 1.5077 | -0.3323 |
| 4 | *COX5B* | 2.1039 | 2.2245 | -0.1853 |
| 5 | *COX7A2* | 4.3592 | 3.5999 | 1.0849 |
| 6 | *NDUFA4* | 4.2201 | 4.9381 | 1.0623 |
| 7 | *NDUFS1* | 3.7547 | 6.9756 | 0.7877 |
| 8 | *NDUFV2* | 2.3177 | 1.3733 | 0.4176 |
| 9 | *UQCRC2* | 3.1839 | 3.0405 | 0.0689 |

**C.** Centroid of each class.

|  | **Genes** | **resistant** | **sensitive** |
| --- | --- | --- | --- |
| 1 | *ATP5C1* | 11.1342 | 10.4905 |
| 2 | *ATP5G3* | 10.227 | 9.8955 |
| 3 | *COX5A* | 8.631 | 8.1074 |
| 4 | *COX5B* | 9.2921 | 9.0064 |
| 5 | *COX7A2* | 9.4777 | 8.7197 |
| 6 | *NDUFA4* | 10.0694 | 9.5515 |
| 7 | *NDUFS1* | 10.8395 | 10.5493 |
| 8 | *NDUFV2* | 9.6369 | 9.0753 |
| 9 | *UQCRC2* | 11.3445 | 10.8657 |
