# Supplementary material for: Insights into gemcitabine resistance in pancreatic cancer: association with metabolic reprogramming and TP53 pathogenicity in patient derived xenografts
Source: J Transl Med. 2024 Aug 5;22:733. doi: 10.1186/s12967-024-05528-6 (PMC11301937; doi:10.1186/s12967-024-05528-6)
Supplement: Supplementary file 5 — Supplementary Material 5: Additional File 5: LASSO model for prediction of binary response based on drug-induced changes. [file 12967_2024_5528_MOESM5_ESM.docx]

**Additional File 5**. LASSO model for prediction of binary response based on drug induced changes. Eighteen genes were selected by LASSO logistic regression. Highlighted genes are also in the LARS model of continuous outcome. Ninety-six percent correctly classified during leave-one-out model cross-validation.

|  | **Symbol** | **Name** | **Coefficient** | **% CV Support** |  |
| --- | --- | --- | --- | --- | --- |
|  |  |  |  |  |  |
| 1 | *A2M* | alpha-2-macroglobulin | 0.10958 | 60.71 |  |
| 2 | *ALDOA* | aldolase, fructose-bisphosphate A | -1.8177 | 100 |  |
| 3 | *BGN* | biglycan | 0.27798 | 100 |  |
| 4 | *BHLHE40* | basic helix-loop-helix family member e40 | -1.82823 | 100 |  |
| 5 | *CASP9* | caspase 9 | -0.47081 | 32.14 |  |
| 6 | *DGKH* | diacylglycerol kinase eta | 3.27652 | 100 |  |
| 7 | *FHL1* | four and a half LIM domains 1 | 0.44423 | 96.43 |  |
| 8 | *FOSB* | FosB proto-oncogene, AP-1 transcription factor subunit | 0.00014 | 50 |  |
| 9 | *HK2* | hexokinase 2 | -0.10642 | 71.43 |  |
| 10 | *IFI27* | interferon alpha inducible protein 27 | -0.20033 | 85.71 |  |
| 11 | *KRT19* | keratin 19 | -0.38263 | 64.29 |  |
| 12 | *MREG* | melanoregulin | -0.87828 | 100 |  |
| 13 | *MRPL15* | mitochondrial ribosomal protein L15 | -0.14769 | 46.43 |  |
| 14 | *NRCAM* | neuronal cell adhesion molecule | 0.15217 | 92.86 |  |
| 15 | *POM121* | POM121 transmembrane nucleoporin | 1.70999 | 100 |  |
| 16 | *PRDX1* | peroxiredoxin 1 | -0.32627 | 39.29 |  |
| 17 | *TIMM13* | translocase of inner mitochondrial membrane 13 | -0.50519 | 53.57 |  |
| 18 | *UXT* | ubiquitously expressed prefoldin like chaperone | -0.34199 | 60.71 |  |
