# Supplementary material for: Insights into gemcitabine resistance in pancreatic cancer: association with metabolic reprogramming and TP53 pathogenicity in patient derived xenografts
Source: J Transl Med. 2024 Aug 5;22:733. doi: 10.1186/s12967-024-05528-6 (PMC11301937; doi:10.1186/s12967-024-05528-6)
Supplement: Supplementary file 6 — Supplementary Material 6: Additional File 6: Least Angle Regression model for prediction of continuous response based on drug-induced gene expression changes. [file 12967_2024_5528_MOESM6_ESM.docx]

**Additional File 6.** Least Angle Regression (LARS) for prediction of continuous response based on drug induced gene expression changes. Twenty-six genes were selected by LARS. Highlighted genes are also in the LASSO regression model of binary outcome. The prediction of new samples can be calculated by the formula: $\sum_{i} c_{i}x_{i}+0.572$, where *c_i_* and *x_i_* are the coefficient and gene expression for the *i-th* gene, respectively. The R^2^ for the model from leave-one-out cross validation is 0.43.

|  | **Symbol** | **Name** | **Coefficient** |  |
| --- | --- | --- | --- | --- |
|  |  |  |  |  |
| 1 | *BHLHE40* | basic helix-loop-helix family member e40 | -0.194 |  |
| 2 | *CPEB2* | cytoplasmic polyadenylation element binding protein 2 | 0.013 |  |
| 3 | *CYP27A1* | cytochrome P450 family 27 subfamily A member 1 | 0.029 |  |
| 4 | *DGKH* | diacylglycerol kinase eta | 0.058 |  |
| 5 | *DHCR7* | 7-dehydrocholesterol reductase | -0.031 |  |
| 6 | *ENO2* | enolase 2 | -0.065 |  |
| 7 | *FHL1* | four and a half LIM domains 1 | 0.087 |  |
| 8 | *HK2* | hexokinase 2 | -0.164 |  |
| 9 | *IFI27* | interferon alpha inducible protein 27 | -0.014 |  |
| 10 | *LDHA* | lactate dehydrogenase A | 0.185 |  |
| 11 | *MREG* | melanoregulin | -0.179 |  |
| 12 | *NRCAM* | neuronal cell adhesion molecule | 0.002 |  |
| 13 | *ORC6* | origin recognition complex subunit 6 | 0.021 |  |
| 14 | *P2RY14* | purinergic receptor P2Y14 | -0.045 |  |
| 15 | *P4HA1* | prolyl 4-hydroxylase subunit alpha 1 | -0.037 |  |
| 16 | *PDK1* | pyruvate dehydrogenase kinase 1 | 0.049 |  |
| 17 | *PLK4* | polo like kinase 4 | -0.015 |  |
| 18 | *POM121* | POM121 transmembrane nucleoporin | 0.16 |  |
| 19 | *RPL36* | ribosomal protein L36 | -0.214 |  |
| 20 | *SLC25A17* | solute carrier family 25 member 17 | -0.043 |  |
| 21 | *SLC30A3* | solute carrier family 30 member 3 | -0.067 |  |
| 22 | *SLCO1A2* | solute carrier organic anion transporter family member 1A2 | -0.008 |  |
| 23 | *TPI1* | triosephosphate isomerase 1 | -0.001 |  |
| 24 | *WNT16* | Wnt family member 16 | -0.008 |  |
| 25 | *WNT6* | Wnt family member 6 | -0.065 |  |
